# Developing a multiomics data-based mathematical model to predict colorectal cancer recurrence and metastasis

**DOI:** 10.1186/s12911-025-03012-9

**Published:** 2025-05-15

**Authors:** Bing Li, Ming Xiao, Rong Zeng, Le Zhang

**Affiliations:** 1 College of Computer Science, Sichuan University, Chengdu, 610065 China; 2 CAS Key Laboratory of Systems Biology, Shanghai Institute of Biochemistry and Cell Biology, Center for Excellence in Molecular Cell Science, Chinese Academy of Sciences, Shanghai, 200031 China; 3 CAS Key Laboratory of Systems Biology, Hangzhou Institute for Advanced Study, University of Chinese Academy of Sciences, Chinese Academy of Sciences, Hangzhou, 310024 China; 4 School of Life Science and Technology, ShanghaiTech University, Shanghai, 201210 China

**Keywords:** Multiomics, Colorectal cancer, Recurrence and metastasis, Data augmentation, Ensemble learning

## Abstract

**Background:**

Colorectal cancer is the fourth most deadly cancer, with a high mortality rate and a high probability of recurrence and metastasis. Since continuous examinations and disease monitoring for patients after surgery are currently difficult to perform, it is necessary for us to develop a predictive model for colorectal cancer metastasis and recurrence to improve the survival rate of patients.

**Results:**

Previous studies mostly used only clinical or radiological data, which are not sufficient to explain the in-depth mechanism of colorectal cancer recurrence and metastasis. Therefore, this study proposes such a multiomics data-based predictive model for the recurrence and metastasis of colorectal cancer. LR, SVM, Naïve-bayes and ensemble learning models are used to build this predictive model.

**Conclusions:**

The experimental results indicate that our proposed multiomics data-based ensemble learning model effectively predicts the recurrence and metastasis of colorectal cancer.

**Supplementary Information:**

The online version contains supplementary material available at 10.1186/s12911-025-03012-9.

## Background

Colorectal cancer is the fourth most deadly cancer worldwide [1]. Although therapies for colorectal cancer keep improving, the mortality rate remains high. Since cancer metastasis is the most important cause of death of patients with colorectal cancer [1–3], the metastasis status is a very important indicator for the clinical treatment of colorectal cancer.

Surgery is the main clinical treatment used currently, but patients who undergo colorectal cancer resection still have a high probability of developing recurrence and metastasis [4]. Moreover, the postoperative recurrence and metastasis status will continue to affect the disease status and survival time after surgery. Currently, continuous examinations and disease monitoring for patients after surgery are very difficult to conduct. Thus, if we can develop such a mathematical model that predicts postoperative metastasis in patients, we will be able to monitor high-risk patients and provide targeted interventions and precise medical treatments to significantly improve the survival rate of patients.

Previously, most colorectal cancer recurrence and metastasis studies manually select the key features [5–13] from a single omics dataset using various data mining methods, such as correlation coefficient test, chi-square test, t test or Mann-Whitney U test [8, 14–20], resulting in subjectivity and inconsistencies of the selected features. Because our multiomics datasets described in the data source section consist of not only clinical and somatic mutation data but also high-dimensional proteomics (6400 dimensions) and phosphoproteomics (22,000 dimensions) data, our first research question is how to develop such a feature selection and high dimensionality reduction algorithm that processes these high-dimensional multiomics colorectal cancer datasets.

Previous studies usually employed radiological data [14–16], clinical data [5–7, 17] or gene expression data [8, 9] to investigate the recurrence and metastasis of colorectal cancer. However, the occurrence and development of colorectal cancer recurrence and metastasis are so complicated [21] that the use of radiological, clinical or gene expression data alone is not sufficient to comprehensively and deeply explain the mechanism underlying the recurrence and metastasis of colorectal cancer. Recently, Chen Li et al. reported that the analysis of proteomics and phosphoproteomics data from the primary tumour alone successfully identifies metastatic cases [22, 23]. Since the collection of large amounts of multiomics data to optimize the weight of the classifiers of the model used to predict the recurrence and metastasis of colorectal cancer is very expensive and time-consuming, our second research question is how to employ a computational algorithm to perform data augmentation for colorectal cancer predictions.

Also, previous studies have usually employed a data mining algorithm [24–32], such as Cox [6, 8, 11, 12, 33, 34], logistic regression [5, 14, 16, 17], decision tree [17, 35–37] and random forest [15], to model the recurrence and metastasis of colorectal cancer. However, since the predictive accuracy for different omics data is sensitive to the data mining algorithm, the use of a single model does not take advantage of multiomics data to increase the predictive power. Therefore, our third research question is how to build such a predictive model that takes advantage of multiomics data and results in a high predictive accuracy for the recurrence and metastasis of colorectal cancer.

To answer our research questions, this study proposes the following three innovations to determine the recurrence and metastasis of colorectal cancer. First, we integrated multiple statistical tests to select the key features from a multiomics dataset. Second, we employed data augmentation to increase the size of the dataset for model training. Third, we built an ensemble learning model [38, 39] to increase the predictive accuracy.

Next, based on the three innovations listed above, we propose our research plan as described below. First, we integrated Student’s t test, Mann-Whitney U test, ANOVA (Analysis of Variance), chi-square test, and Fisher’s exact test [40–46] to select the key features from clinical, somatic mutation, proteomics, and phosphoproteomics datasets and then employed PCA (principal component analysis) [47, 48] to perform dimensional reduction. Second, we conducted data augmentation using the SMOTE algorithm to increase the dataset size for model training. Third, we integrated the logistic regression (LR), support vector machine (SVM), and Naive-Bayes algorithms to build an ensemble learning predictive model for the recurrence and metastasis of colorectal cancer.

At last, we selected 3 key features from clinical data, 3 key features from somatic mutations, 89 key features from proteomics and 15 key features from phosphoproteomics. Afterward, we performed dimensional reduction for proteomics and phosphoproteomics features to obtain two principal components. After data augmentation, the sample size increased from 144 to 288, which met the requirement of model training. Finally, we developed a novel multiomics databased ensemble learning model for the prediction of recurrence and metastasis of colorectal cancer that outperformed the classical LR, Naive-Bayes, and SVM models.

## Methods

### Data source

Our research data were obtained from our previous study [22], which were originally collected from 146 patients with colorectal cancer at Shanghai Hospital, China [22]. Our research data consisted of clinical (clinicopathologic features and prognosis information), somatic mutations (information on somatic single-nucleotide variants (SNVs) and small insertions-deletions (INDELs) identified by WES), proteomics (6,408 quantified protein expression data that were subjected to median normalization by column and log2 transformation) and phosphoproteomics data (22,000 quantified phosphoprotein expression data that were subjected to median normalization by column and log2 transformation). Among the 146 patients, 70 experienced recurrence and metastasis after surgery and were labelled with one; 74 patients were free from recurrence and metastasis and were labelled with zero; and 2 patients lacked the label. Thus, only samples from 144 patients were used in our study. The informed consent was obtained from all subjects. The experimental protocol was approved by Shanghai Changhai Hospital Ethics Committee (CHEC2017-235, Shanghai, China) [22].

### Workflow of the study

**Fig. 1:**
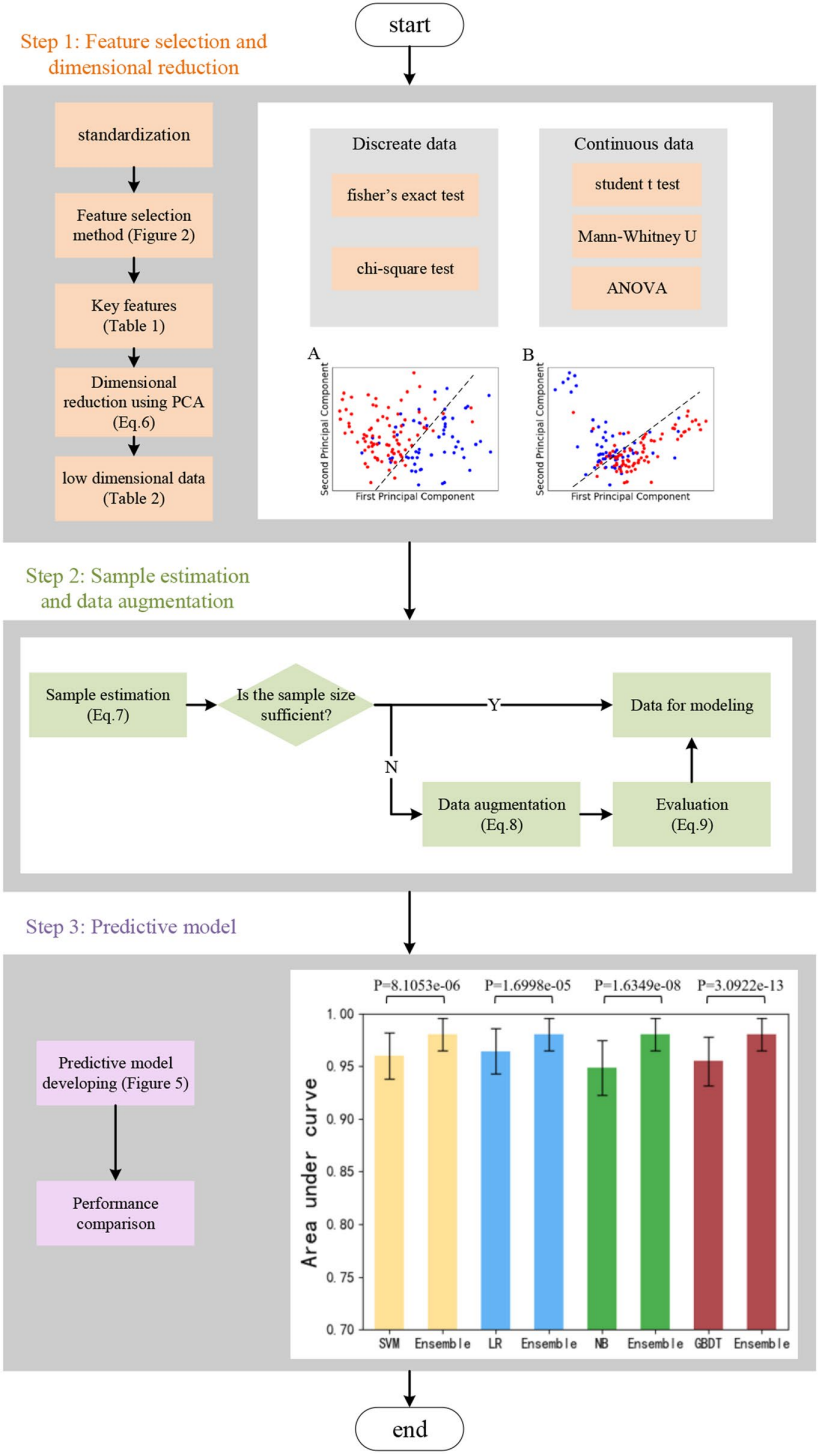
Workflow of the study. The P values in Step 3 were calculated using the T test [61]

Figure 1 describes the workflow of the study. First, we selected the key features from all datasets and then employed PCA to perform dimensional reduction. Next, we conducted data augmentation to increase the sample size for model training. Finally, we integrated the LR, SVM, and Naive-Bayes algorithms to develop an ensemble learning model for colorectal cancer recurrence and metastasis.

### Details for feature selection

#### Fisher’s exact test

Construct a contingency table.

**Table:** 

	A-positive	A-negative	Total
B-positive	a	b	a + b
B-negative	c	d	c + d
total	a + c	b + d	n

1 $$\:\begin{array}{c}p=\frac{{C}_{a+b}^{a}{C}_{c+d}^{c}}{{C}_{n}^{a+c}}=\frac{\left(a+b\right)!\left(c+d\right)!\left(a+c\right)!\left(b+d\right)!}{a!b!c!d!n!}\end{array}$$

#### Chi-square test

2 $$\:\begin{array}{c}{\chi}^{2}=\sum_{i=1}^{k}\frac{{\left({x}_{i}-n{p}_{i}\right)}^{2}}{n{p}_{i}}\end{array}$$

Here, $\:n$ is the number of observations, $\:k$ is the number of different classes, $\:{x}_{i}$ is the observed value and $\:{p}_{i}$ is the probability of class $\:i$.

#### Student’s t test

3.1 $${t = \frac{{{\overline X_1} - {\overline X_2}}}{{{s_p}\sqrt {\frac{1}{{{n_1}}} + \frac{1}{{{n_2}}}} }}}$$

3.2 $$\:\begin{array}{c}{s}_{p}=\sqrt{\frac{\left({n}_{1}-1\right){{s}^{2}}_{{X}_{1}}+\left({n}_{2}-1\right){{s}^{2}}_{{X}_{2}}}{{n}_{1}+{n}_{2}-2}}\end{array}$$

Here, $\:{{s}^{2}}_{{X}_{1}}$ and $\:{{s}^{2}}_{{X}_{2}}$ are the variances of the two sets and n is the size of the set.

#### Mann-Whitney U test

4.1 $$\:\begin{array}{c}U=\sum_{i=1}^{n}\sum_{j=1}^{m}S\left({X}_{i},{Y}_{j}\right)\end{array}$$

4.2 $$\:\begin{array}{c}S\left({X}_{i},{Y}_{j}\right)=\left\{\begin{array}{c}1\:\:\:\:\:\:\:\:\:\:Y<X\\\:0.5\:\:\:\:\:\:Y=X\\\:0\:\:\:\:\:\:\:\:\:\:Y>X\end{array}\right.\end{array}$$

#### ANOVA

$$\:\begin{array}{c}{SS}_{total}={SS}_{treatment}+{SS}_{error}\end{array}$$

$$\:\begin{array}{c}{DF}_{total}={DF}_{treatment}+{DF}_{error}\end{array}$$

$$\:\begin{array}{c}{MS}_{treatment}={SS}_{treatment}/{DF}_{treatment}\end{array}$$

$$\:\begin{array}{c}{MS}_{error}={SS}_{error}/{DF}_{error}\end{array}$$

5 $$\:\begin{array}{c}F=\frac{{MS}_{treatment}}{{MS}_{error}}=\frac{{SS}_{treatment}/{DF}_{treatment}}{{SS}_{error}/{DF}_{error}}\end{array}$$

Here, $\:SS$ represents the sum of squares, $\:DF$ represents the degree of freedom and $\:MS$ is the mean squares.

## Results

### Feature selection and dimensional reduction

To answer the first research question, we propose a feature selection and dimensional reduction workflow to process the multiomics data as described below.

### Feature selection

**Fig. 2:**
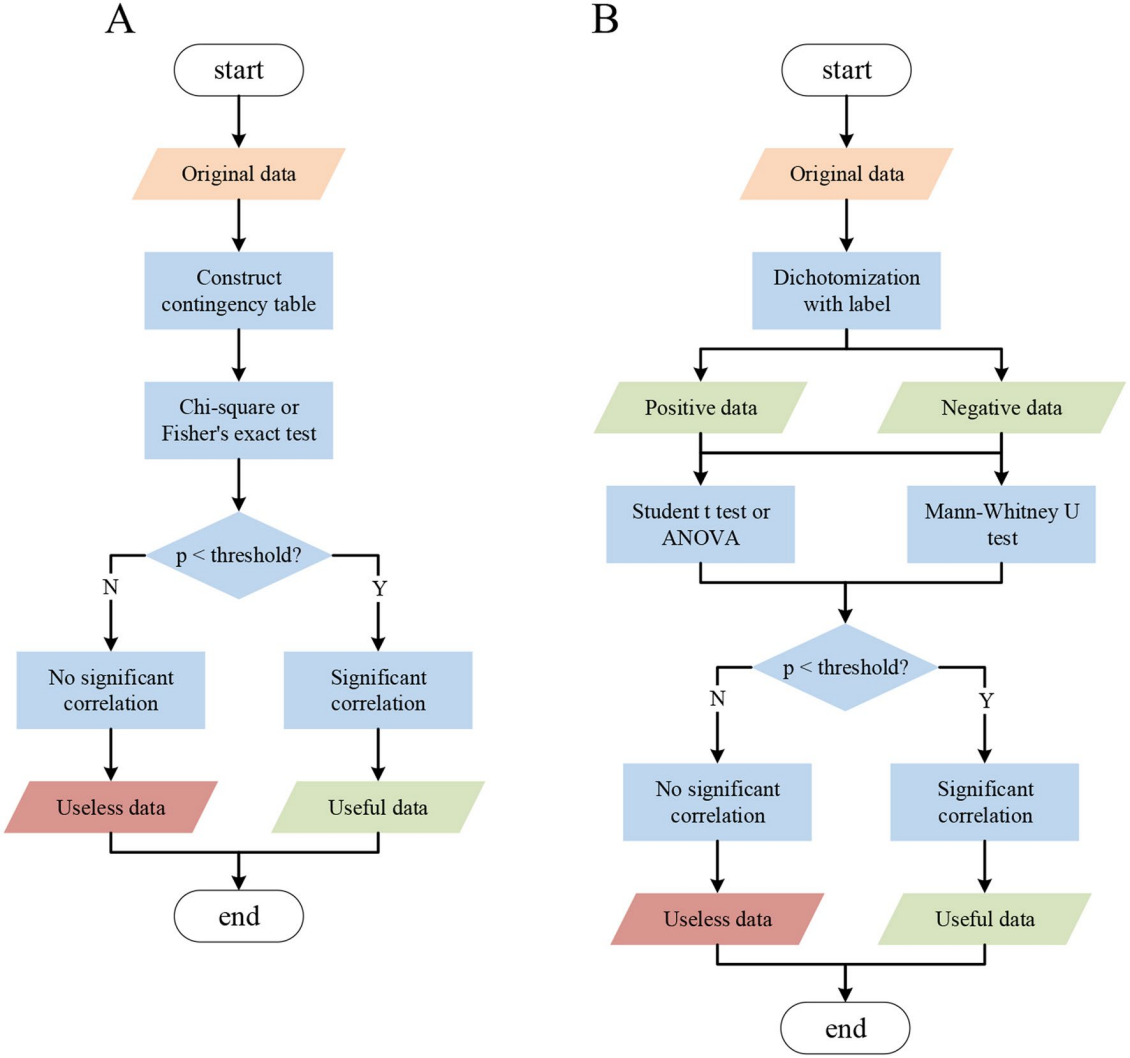
Feature selection methods for (**A**) discrete and (**B**) continuous datasets

We proposed a robust feature selection method for multiomics data, and Fig. 2 illustrates two feature selection methods for discrete and continuous data. For discrete data, we used Fisher’s exact test (Eq. 1) or the chi-square test (Eq. 2) [22] to determine the correlations between each feature and their label (Fig. 2A). For continuous data, we divided the dataset into two datasets according to the label, and then we integrated Student’s t test (Eq. 3) [17, 49], Mann-Whitney U test (Eq. 4) [15] and ANOVA (Eq. 5) [50] to perform feature selection [10, 13, 35, 48, 51, 52, 53, 54] (Fig. 2B). Key equations are listed in Methods.

Table 1 lists the key features for each dataset, and Supplementary Table S1 describes the feature selection procedure.

**Table 1:** The key features of each dataset

Dataset	Features
Clinical data	Lymph node, Metastasis, Calcium nodus
Somatic mutations	COL6A3, OTOG, KAL1
Proteomics	A0A024R046, A0A024R056, A0A024R0Y5, A0A024R1S8, A0A024R2U7, A0A024R3B5, A0A024R5K1, A0A024R7I3, A0A024R9G4, A0A024RCX8, A0A024RCY1, A0A087WTA8, A0A0A0MRF6, A0A0A0MSM0, A0A0S2Z3J9, A0A140VJC9, A2RUA4, A4D1 × 5, A6NHQ2, A8K2L4, A8K878, B2R4P9, B2R5J1, B2RDF2, B2RDW1, B2ZDQ1, B4DEH0, B7ZB78, C9K0I4, D3DT27, E9PMC9, G1EPM2, O14917, O60493, O75554, P02452, P02461, P02749, P05164, P11908, P14780, P17066, P17600, P18077, P20585, P23378, P24158, P29350, P31947, P35637, P36268, P37802, P40763, P42224, P48061, P49327, P51572, P52209, P52630, P52732, P54707, P63261, P68371, P78346, Q13151, Q13287, Q13884, Q15029, Q2TAM5, Q53SW3, Q546E0, Q6DN03, Q6FIA3, Q7Z434, Q8N8A2, Q8WUM0, Q8WXH0, Q92734, Q96A33, Q96BP3, Q96IS6, Q99497, Q9BTE1, Q9BTT0, Q9NZ08, Q9UI15, Q9UNS2, Q9Y2Z0, Q9Y426
Phosphoproteomics	A0A024R4G1_509_Y, A0A024R4Z6_441_Y, A0A024R9K2_184_S, A0A024RAM4_1817_S, A0A087WVT6_320_S, A0A140VJN8_130_S, B4DPP8_314_T, B4DPP8_320_S, B4DPP8_325_T, P08670_436_T, P62263_137_S, Q6WKZ4_206_S, Q6WKZ4_338_S, Q8IZ21_358_T, Q92625_628_S

### Dimensional reduction

Since Table 1 shows that the features of proteomics and phosphoproteomics data still had high dimensions, we carried out PCA (Eq. 6) to reduce the dimensions of these two datasets [55].

6 $$\:\begin{array}{c}{T}_{L}=\:X{W}_{L}\end{array}$$

In Eq. 6, $\:{W}_{L}$ maps the original data $\:X$ with $\:p$ variables to a new space $\:T$ with $\:p$ variables that are uncorrelated over the dataset, and only the first $\:L$ principal components are retained after dimensional reduction.

**Fig. 3:**
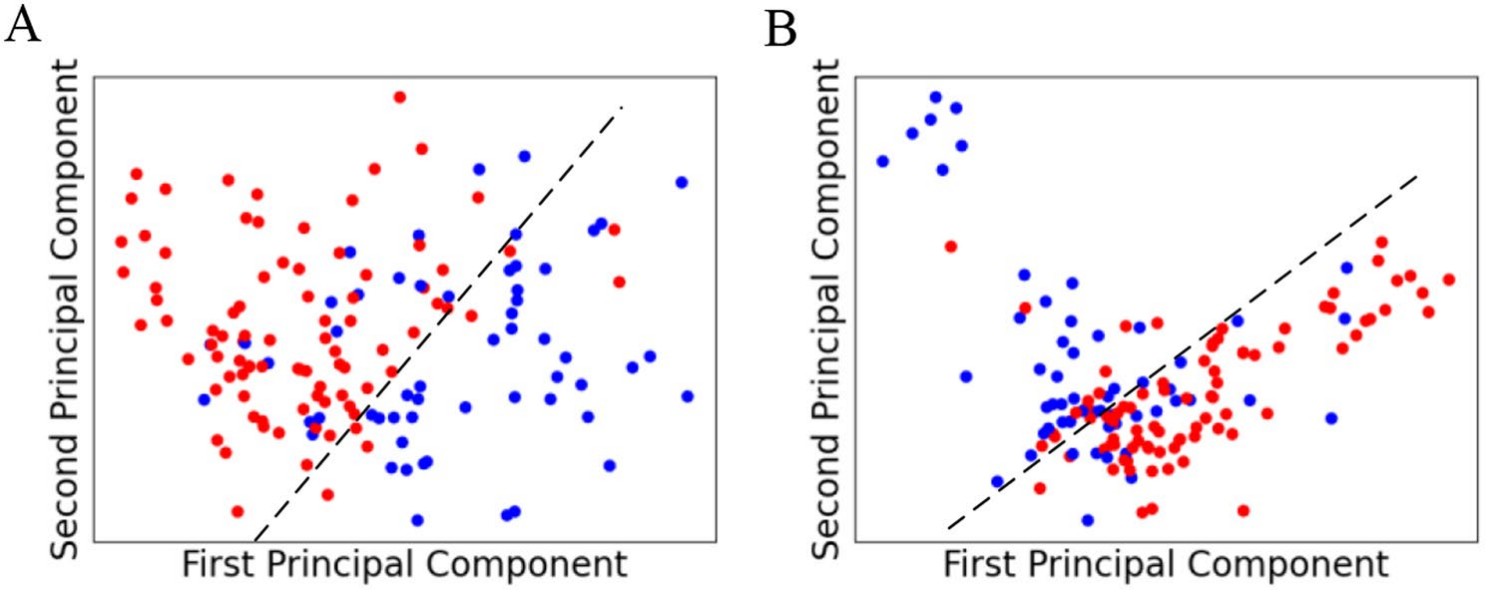
Illustration of the first two principal components. Here, red points represent patients without recurrence and metastasis, and blue points represent patients with recurrence and metastasis. (**A**) Proteomics data and (**B**) phosphoproteomics data

Figure 3 shows the classification results when we chose the first two principal components for dimensional reduction. Since the first two principal components successfully segmented patients with recurrence and metastasis (blue) and patients without recurrence and metastasis (red), we chose the first two principal components to reduce dimensions for proteomics (Fig. 3A) and phosphoproteomics data (Fig. 3B).

After dimensional reduction, the number of features of the clinical data, somatic mutations, proteomics and phosphoproteomics datasets decreased from 110 to 11. Table 2 lists the final features of each dataset, and Supplementary Table S2 describes the dimensional reduction procedure.

**Table 2:** The results of dimensional reduction

Dataset	Features
Clinical data	Lymph node, Metastasis, Calcium nodus
Somatic mutations	COL6A3, OTOG, KAL1
Proteomics	First two principal components
Phosphoproteomics	First two principal components

### Data augmentation

To answer our second question, we used the results of feature selection and dimensional reduction as input (Tables 1 and 2) to estimate if the dataset is sufficient large for model training. If the dataset size was insufficient, we employed data augmentation to increase the dataset size using the method described below.

### Sample Estimation

We employed Eq. 7 to compute the optimum sample size (n) for each selected feature with respect to the preset statistical significance [56, 57].

7 $$\:\begin{array}{c}n=\frac{{\sigma\:}^{2}\left({{Q}_{1}}^{-1}+{{Q}_{2}}^{-1}\right){\left({\mu}_{\alpha}+{\mu}_{\beta}\right)}^{2}}{{\delta}^{2}}\end{array}$$

Here, σ is the standard deviation; $\:{\mu\:}_{\alpha\:}$ and $\:{\mu\:}_{\beta\:}$ are the critical values of the U-test at the first type of error rate and the second type of error rate; $\:{Q}_{1}$ and $\:{Q}_{2}$ are the proportions of each part of the population after the dichotomization procedure; and $\:\delta\:$ is the difference between the mean of the two datasets.

After performing sample size estimation for every selected feature in Table 1, the optimum sample size n of each dataset is listed in Table 3. Because our original dataset only consisted of 144 labelled data (2.1 Data source section), the dataset was smaller than the optimum sample size n of some datasets (Table 3), indicating that our samples were not sufficient for model training.

**Table 3:** Optimum sample size N of each dataset

Dataset	Estimated sample size
Clinical data	86
Somatic mutations	231
Proteomics	142
Phosphoproteomics	157

### SMOTE algorithm

The SMOTE algorithm (Eq. 8) [58] was previously used for oversampling. Here, we employed it for data augmentation. The procedure and key equation are listed below.

#### Input

Dataset $\:T=\:\left\{\:\right({x}_{1},\:{y}_{1}),\:({x}_{2},\:{y}_{2}),\:\dots\:,\:({x}_{n},\:{y}_{n}\left)\:\right\}$, where $\:{x}_{i}$ is the examples and $\:{y}_{i}$ is the labels; number of samples $\:m$; number of nearest neighbours $\:k$.

**Process**:

For each data $\:({x}_{i},\:{y}_{i})$ in $\:T$:

Find $\:k$ nearest neighbours with the same label.

$$\:K=\left\{\:\right({x}_{i1},\:{y}_{i}),\:({x}_{i2},\:{y}_{i}),\:\dots\:,\:({x}_{ik},\:{y}_{i}\left)\:\right\}$$

Choose $\:m$ neighbours randomly in $\:K$.

$$\:M=\left\{\:\right({x}_{i1},\:{y}_{i}),\:({x}_{i2},\:{y}_{i}),\:\dots\:,\:({x}_{im},\:{y}_{i}\left)\:\right\}$$

For each data $\:({x}_{ij},\:{y}_{ij})$ in $\:M$:

8 $$\:\begin{array}{c}{x}_{new}={x}_{i}+rand\left(\text{0,1}\right)*\left({x}_{ij}-{x}_{i}\right)\end{array}$$

#### Output

Generated new dataset $\:G$ with label$\:\:{y}_{i}$

$$\:G=\left\{\:\right({x}_{1},\:{y}_{i}),\:({x}_{2},\:{y}_{i}),\:\dots\:,\:({x}_{n*m},{y}_{i}\left)\right\}$$

We used the SMOTE algorithm to augment the data with pseudo dataset generation by setting $\:m=1$ and $\:k=5,$ as described in detail in Supplementary Table S3. Then, the sample size increased from 144 (original dataset) to 288 (pseudo dataset). Since the size of the pseudo dataset (288) was greater than estimated sample size (231), we consider that it meets the requirement for the sample estimation.

### Evaluation of the pseudo dataset quality

We employed the maximum Fisher’s discriminant ratio or F1 [59] to validate whether the generated dataset was sufficient for classification and to evaluate the quality of the data augmentation process for the pseudo dataset, as described in a previous study [58]. The F1 value calculated using Eq. 9 shows the degree of overlap. A high F1 value indicates a low degree of overlap in the datasets, which is better for classification [58].

9.1 $$\:\begin{array}{c}{f}_{i}=\frac{{\left({\mu\:}_{1}-{\mu\:}_{2}\right)}^{2}}{{{\sigma}_{1}}^{2}+{{\sigma}_{2}}^{2}}\end{array}$$

9.2 $$\:\begin{array}{c}{F}_{1}=\text{max}\left({f}_{i}\right)\end{array}$$

We employed Eq. 9.1 and 9.2 to compute $$\:{f}_{i}$$ for each individual feature $$\:i$$ and F1 value, respectively. $$\:{\mu\:}_{1}$$, $$\:{\mu\:}_{2,}$$$$\:{\sigma\:}_{1}$$, and $$\:{\sigma\:}_{2}$$ are the means and standard errors for the two classes, respectively.

**Fig. 4:**
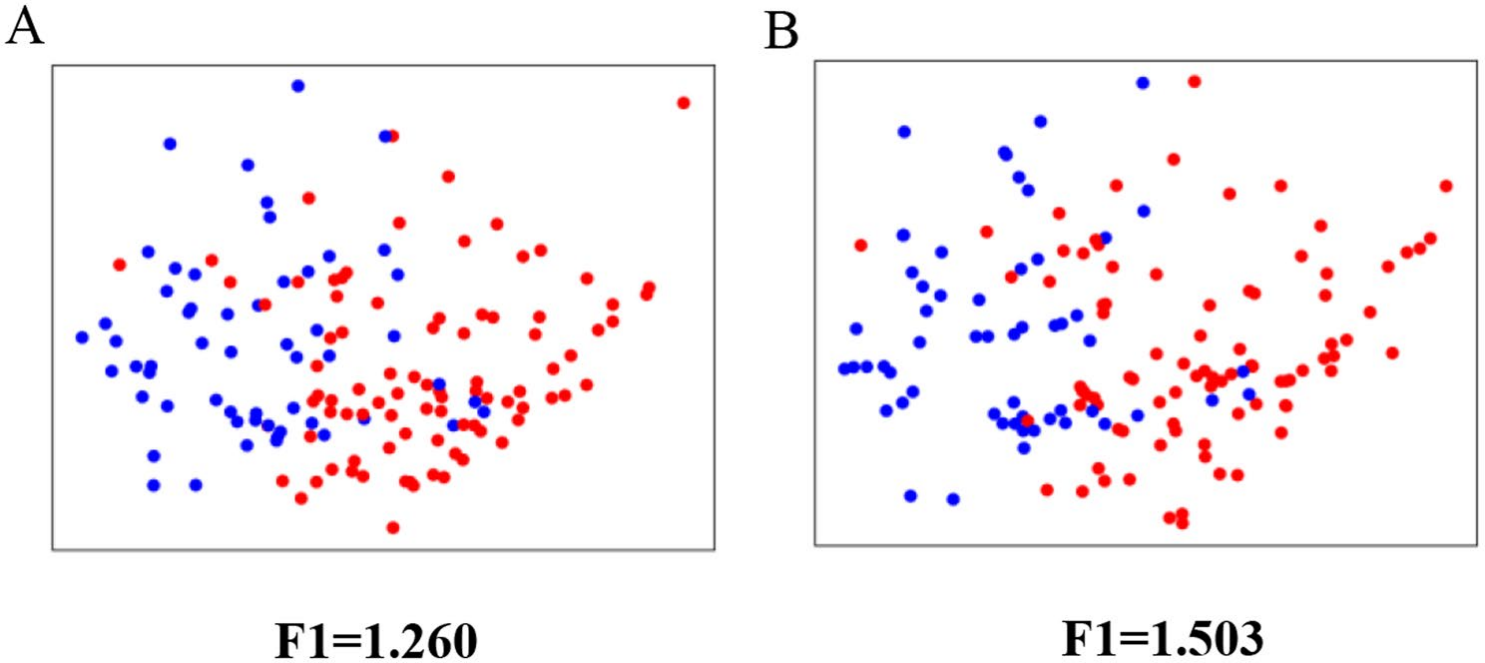
Illustration of the dataset mapped to two dimensions and the F1 value. Here, red points represent patients without recurrence and metastasis, and blue points represent patients with recurrence and metastasis. (**A**) Original dataset and (**B**) generated dataset

As described in a previous study [58], we calculated the F1 value to evaluate the overlap of the two classes. Since the F1 value for the original dataset (Fig. 4A) was less than the F1 value for the SMOTE-generated dataset (Fig. 4B), we consider that the dataset generated by SMOTE has such a lower degree of overlap that is better for classification than the original dataset.

### Predictive model

To answer our third question, we developed an ensemble predictive model using three classical classification methods, the performance of which was measured using K-fold cross validation [12, 18, 51, 52, 57]. The development of the ensemble learning model and comparison of the performance between ensemble learning and classical classification are described below.

### Ensemble learning model development

**Fig. 5:**
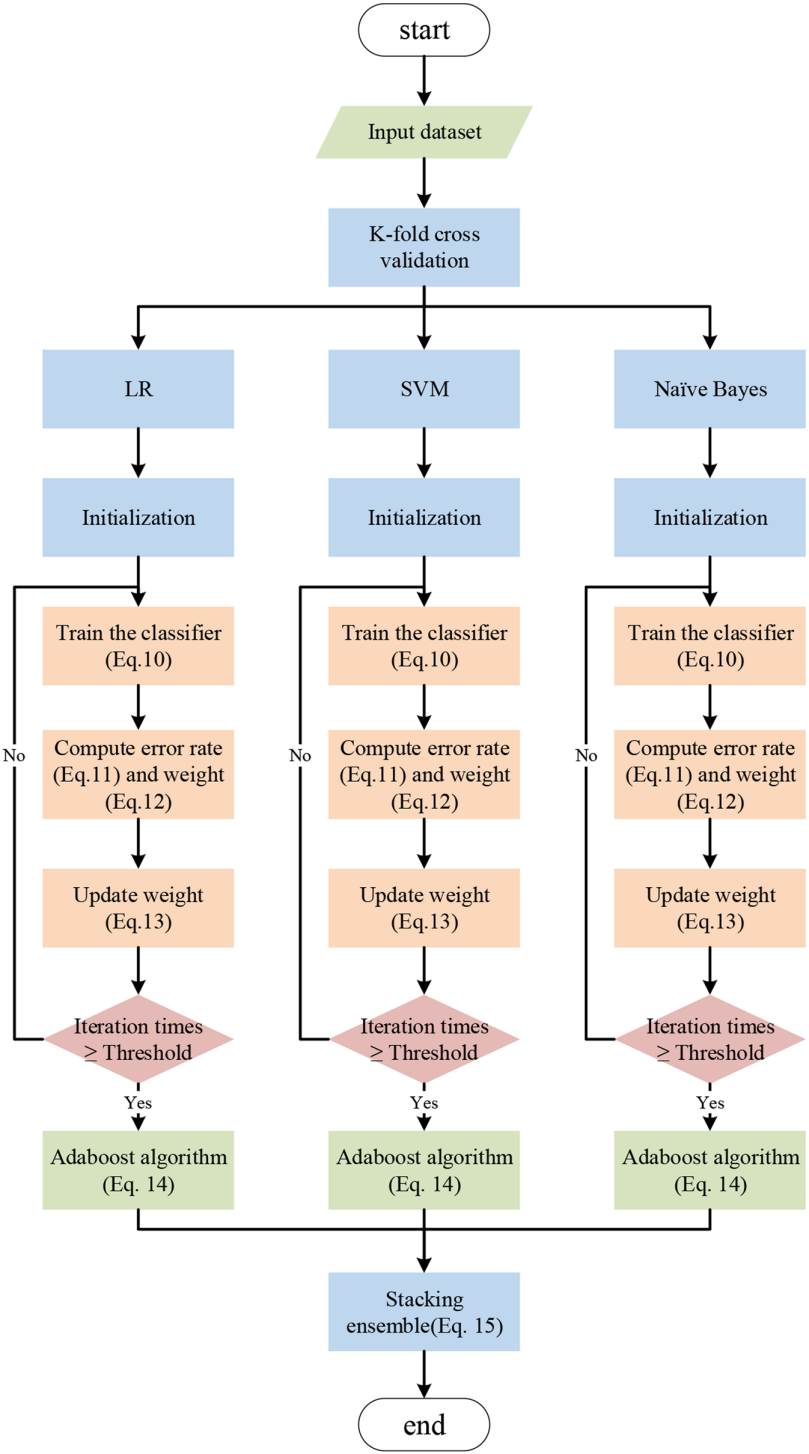
The workflow of ensemble learning model development

Regarding to previous studies [60, 61], we integrate three classical classification methods, LR [62], SVM [63] and Naive-Bayes [64], to develop an ensemble predictive model (Fig. 5) for the recurrence and metastasis of colorectal cancer. The key equations used in this model are listed below.

10 $$\:\begin{array}{c}{D}_{t}\left(i\right)=\frac{1}{n}\end{array}$$

11 $${\varepsilon _t} = \mathop \sum \limits_{\begin{array}{*{20}{c}}{i = 1} \\ {{h_t}\left( {{x_i}} \right) \ne {y_i}} \end{array}}^n {D_t}\left( i \right)$$

12 $$\:\begin{array}{c}{\alpha}_{t}=\frac{1}{2}\text{ln}\left(\frac{1-{\epsilon}_{t}}{{\epsilon}_{t}}\right)\end{array}$$

13 $$\:\begin{array}{c}{D}_{t+1}\left(i\right)=\frac{{D}_{t}}{sum\left({D}_{t+1}\right)}\left\{\begin{array}{c}{e}^{-{\alpha}_{t}}\:{\:\:h}_{t}\left({x}_{i}\right)={y}_{i}\\\:{e}^{{\alpha}_{t}}\:\:\:{\:\:h}_{t}\left({x}_{i}\right)\ne\:{y}_{i}\end{array}\right.\end{array}$$

14 $$\:\begin{array}{c}{H}_{mT}\left(x\right)=\sum_{t=1}^{T}{\alpha}_{t}{h}_{t}\left({x}_{i}\right)\end{array}\left(14\right)$$

15 $$\:\begin{array}{c}{log}\left(\frac{H\left(x\right)}{1-H\left(x\right)}\right)={c}_{0}+\sum_{m=1}^{M=3}{c}_{m}{H}_{mT}\left(x\right)\end{array}$$

Here, $\:{D}_{t}\left(i\right)$ is the weight distribution, $\:t$ is the iteration time, $\:i$ is the index of the sample, and $\:n$ is the number of samples. $\:{\epsilon}_{t}$ and $\:{\alpha}_{t}$ are the error rate and weight of each weak classifier $\:{h}_{t}$, respectively. For a sample set $\:S\:=\:\left\{\:\right({x}_{1},\:{y}_{1}),\:({x}_{2},\:{y}_{2}),\:\dots\:,\:({x}_{n},\:{y}_{n}\left)\:\right\}$, $\:{x}_{n}$ represents the samples and $\:{y}_{n}\in\:\:\{0,\:1\}$ represents the labels; $\:{y}_{i}=0$ indicates that $\:{x}_{i}$ is not a patient with recurrence and metastasis, and $\:{y}_{i}=1$ indicates that $\:{x}_{i}$ is a patient with recurrence and metastasis. $\:{H}_{mT}$ is the homomorphic integration for each weak classifier $\:{h}_{t}$; $\:m$ is the index of the weak classifier, $\:m\:=\:1,\:2,\:3$; $\:T$ is the threshold of the iteration time; $\:H\left(x\right)$ is the ensemble classifier; and $\:{c}_{m}$ is the weight of each weak classifier.

### Predictive performance comparison

**Fig. 6:**
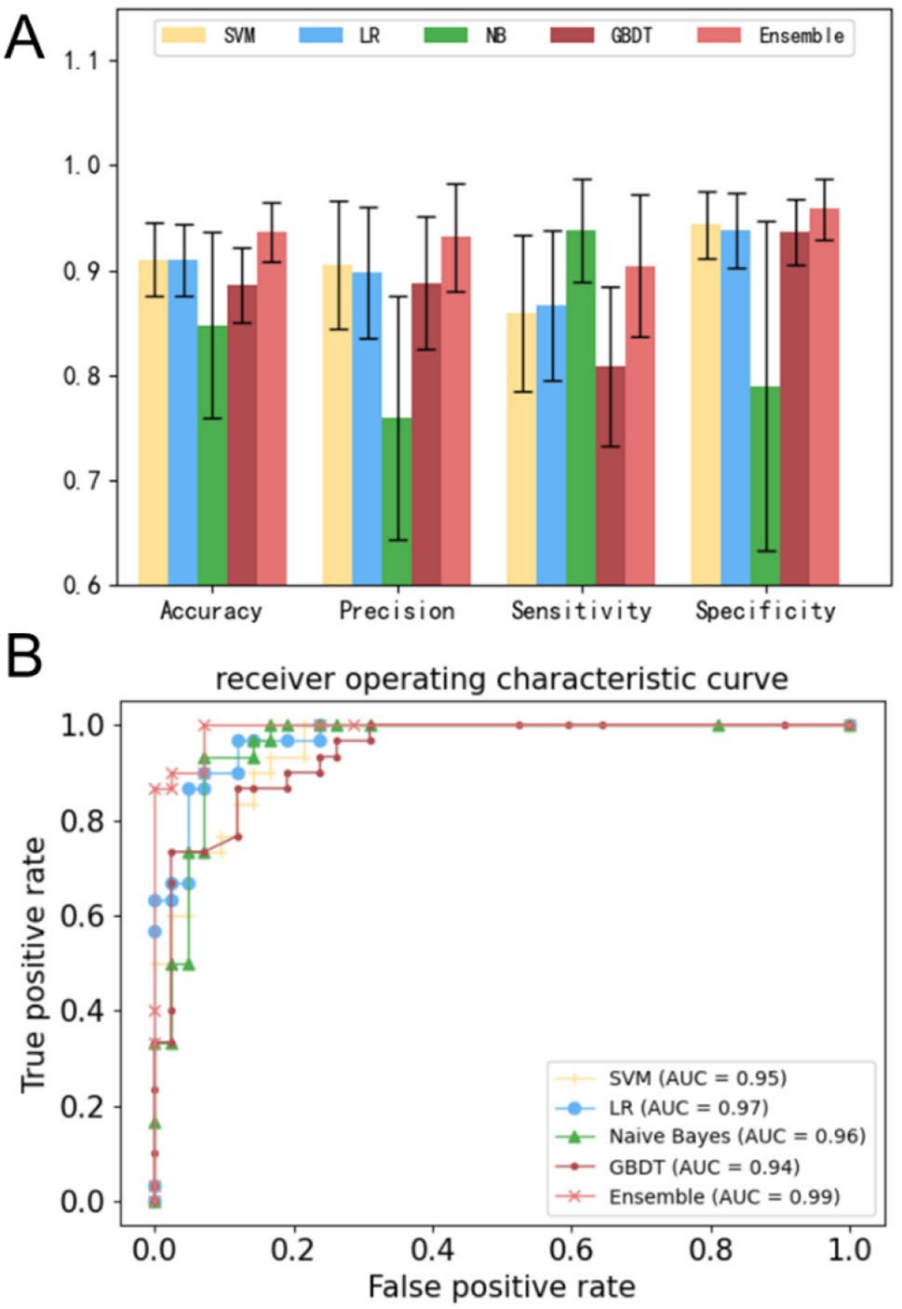
Model performance. (**A**) Comparison of the classification performance of LR, SVM, Naive-Bayes, and ensemble learning models; (**B**) ROC curves plotted for LR, SVM, Naive-Bayes, and ensemble learning models

Figure 6A compares the classification performance for the LR, Naive-Bayes, SVM, GDBT, and ensemble learning models based on four commonly used classification measurements (Supplementary Table S4) [60]. Supplementary Table S5 lists the means and standard deviations for the results presented in Fig. 6A. Supplementary Table S6 lists the P values for LR, NB, SVM, GDBT and ensemble learning models. Figure 6 and Supplementary Tables S5 and S6 show the statistically significantly better classification performance of the ensemble learning model than that of the other four models. Figure 6B shows that if we comprehensively consider both sensitivity and specificity by constructing ROC curves [33], the ROC curve of the ensemble learning model is better than that of LR, Naive-Bayes, SVM and GDBT models.

## Discussion

This study aimed to develop a multiomics data-based mathematical model to predict the recurrence and metastasis of colorectal cancer by answering three scientific questions.

To answer the first question, we used multiple data mining methods with the pipelines illustrated in Fig. 2 to explore the key features and employed PCA to reduce the dimensions of those features. Since Table 1 shows not only the selected features with statistically significant differences between positive and negative classes but also manually reviewed evidence indicating that COL6A3 [65] and TNM [66] are related to the development of colorectal cancer, OTOG [67] and KAL1 [68] are related to gastric cancer and oral squamous cell carcinoma, and most of the functions of proteomics and phosphoproteomics features [69] are related to cancer, we consider that these features can be employed as classifiers for our proposed predictive model. Moreover, since Fig. 3 shows that the positive and negative classes were successfully distinguished from each other, we consider that our dimensional reduction is efficient.

To answer the second question, we employed data augmentation to generate the pseudo dataset for model training (Table 3). After data augmentation, we calculated the F1 value [58] to evaluate the quality of the pseudo dataset. As shown in Fig. 4, the pseudo dataset generated by the SMOTE algorithm has a greater F1 value than the original dataset, indicating that the pseudo dataset not only meets the requirement of sample estimation but also ensures the data quality and robustness. Although SMOTE was the most used and effective method for numerical data augmentation, we have also tried other data augmentation methods, such as adding noise to create new data [70], but experiments showed that the data created by this method was not good enough (Shown in Figure S1, F1 value for different methods: Original: 1.260, SMOTE: 1.503, Noise: 1.259). As we explained above, the greater the F1 value, better quality of the generated data. So, we can see the quality of SMOTE is better than other data augmentation methods.

To answer the third question, we developed an ensemble learning predictive model for the recurrence and metastasis of colorectal cancer. Figure 6 and Supplementary Table S6 show the significantly better performance of the ensemble model than the single classical machine learning model. However, Fig. 6A shows that the sensitivity of the ensemble learning model is not better than that of the Naïve bayes method. A potential explanation is that the ensemble learning model employs accuracy as the objective function to optimize the key weights (Eqs. 12 and 13) for each weak classifier, and thus it does not exhibit the best performance for the other three measurements, especially for sensitivity. On the other hand, Fig. 6B shows that the ROC curves of ensemble learning are better than those of the other three models, implying that the ensemble model still performs better than the single classical machine learning model if we comprehensively consider both sensitivity and specificity.

## Conclusion

This study developed a multiomics data-based mathematical model to predict the recurrence and metastasis of colorectal cancer. First, we develop a feature selection and high dimensionality reduction algorithm that processes these high-dimensional multiomics colorectal cancer datasets. Second, we employ a computational algorithm to perform data augmentation for colorectal cancer prediction. Third, we build a predictive model that takes advantage of multiomics data and results in a high predictive accuracy for the recurrence and metastasis of colorectal cancer.

Although we have already achieved substantial progress in predicting colorectal cancer recurrence and metastasis, the unclear connections between proteomics and phosphoproteomics data remain to be solved. Thus, we will integrate more multiomics data and advanced bioinformatics methods into the current predictive model to increase its predictive power in the distant future.

## Electronic supplementary material

Below is the link to the electronic supplementary material.

Supplementary Material 1: Additional file 1– Supplementary Material: Supplementary information for the Supplementary Tables S1-S6, Figure S1 and code availability

Supplementary Material 2: Additional file 2– Supplementary Table S1: The detailed results of feature selection (the p values) are listed in Supplementary Table S1

Supplementary Material 3: Additional file 3– Supplementary Table S2: The results of dimensional reduction are listed in Supplementary Table S2

Supplementary Material 4: Additional file 4– Supplementary Table S3: The results of data augmentation are listed in Supplementary Table S3

## Data Availability

The dataset supporting the conclusions of this article is available in the https://ars.els-cdn.com/content/image/1-s2.0-S153561082030413X-mmc2.xlsx.

## Abbreviations

LR: Logistic Regression
SVM: Support Vector Machine
NB: Naive-Bayes
ANOVA: Analysis of Variance
PCA: Principal Component Analysis
SMOTE: Synthetic Minority Oversampling Technique
SNVs: Single-Nucleotide Variants
INDELs: Insertions-Deletions
WES: Whole-Exome Sequencing
ROC: Receiver Operating Characteristic Curve
