# Supplementary material for: Developing a multiomics data-based mathematical model to predict colorectal cancer recurrence and metastasis
Source: BMC Med Inform Decis Mak. 2025 May 15;25(Suppl 2):188. doi: 10.1186/s12911-025-03012-9 (PMC12082861; doi:10.1186/s12911-025-03012-9)
Supplement: Supplementary file 1 — Supplementary Material 1: Additional file 1– Supplementary Material: Supplementary information for the Supplementary Tables S1-S6, Figure S1 and code availability [file 12911_2025_3012_MOESM1_ESM.docx]

Supplementary Material

1. Supplementary Tables

**Table S1. The p values of feature selection**

The detailed results of feature selection (the p values) are listed in Table S1.xlsx

**Table S2. Data after dimensional reduction**

The results of dimensional reduction are listed in Table S2.xlsx.

**Table S3. Pseudo dataset generated by SMOTE**

The results of data augmentation are listed in Table S3.xlsx.

**Table S4. Model performance indicator**

| Index | Formula | Illustration |
| --- | --- | --- |
| Accuracy | $\frac{TP+TN}{P+N}$ | TP: actual positive and is recognized as positive |
| Precision | $\frac{TP}{TP+FP}$ | TN: actual negative and is recognized as negative |
| Sensitivity | $\frac{TP}{TP+FN}$ | FP: actual negative and is recognized as positive |
| Specificity | $\frac{TN}{FP+TN}$ | FN: actual positive and is recognized as negative |

**Table S5. The result of predictive performance comparison**

| AUC | SVM | LR | Naive Bayes | GBDT | Ensemble |
| --- | --- | --- | --- | --- | --- |
| Accuracy | 0.9102±0.0348 | 0.9095±0.0345 | 0.8474±0.0885 | 0.8858±0.0353 | 0.9368±0.0285 |
| Precision | 0.9050±0.0612 | 0.8976±0.0627 | 0.7591±0.1159 | 0.8881±0.0629 | 0.9315±0.0511 |
| Sensitivity | 0.8589±0.0746 | 0.8669±0.0714 | 0.9385±0.0493 | 0.8088±0.0764 | 0.9043±0.0680 |
| Specificity | 0.9437±0.0317 | 0.9379±0.0357 | 0.7899±0.1569 | 0.9367±0.0318 | 0.9586±0.0290 |

**Table S6. The p values of T test on AUROC**

| Model | AUROC | P value |
| --- | --- | --- |
| Ensemble | 0.9802±0.0155 | 6.3068e-10 |
| SVM | 0.9599±0.0222 |  |
| Ensemble | 0.9802±0.0155 | 4.4825e-07 |
| LR | 0.9643±0.0216 |  |
| Ensemble | 0.9802±0.0155 | 8.8430e-16 |
| NB | 0.9488±0.0261 |  |
| Ensemble | 0.9802±0.0155 | 3.0922e-13 |
| GBDT | 0.9548±0.0233 |  |

1. Supplementary Figure

**Figure S1.** Illustration of the dataset mapped to two dimensions and the F1 value with different data augmentation methods. Here, red points represent patients without recurrence and metastasis, and blue points represent patients with recurrence and metastasis.

1. Code

Code used for predictive model is available at:

<https://github.com/skytea/CRC>
